# PPM1D controls nucleolar formation by up-regulating phosphorylation of nucleophosmin

**DOI:** 10.1038/srep33272

**Published:** 2016-09-13

**Authors:** Yuuki Kozakai, Rui Kamada, Junya Furuta, Yuhei Kiyota, Yoshiro Chuman, Kazuyasu Sakaguchi

**Affiliations:** 1 Laboratory of Biological Chemistry, Department of Chemistry, Faculty of Science, Hokkaido University, Sapporo, Japan

## Abstract

An increase of nucleolar number and size has made nucleoli essential markers for cytology and tumour development. However, the underlying basis for their structural integrity and abundance remains unclear. Protein phosphatase PPM1D was found to be up-regulated in different carcinomas including breast cancers. Here, we demonstrate for the first time that PPM1D regulates nucleolar formation via inducing an increased phosphorylation of the nucleolar protein NPM. We show that PPM1D overexpression induces an increase in the nucleolar number regardless of p53 status. We also demonstrated that specific sequential phosphorylation of NPM is important for nucleolar formation and that PPM1D is a novel upstream regulator of this phosphorylation pathway. These results enhance our understanding of the molecular mechanisms that govern nucleoli formation by demonstrating that PPM1D regulates nucleolar formation by regulating NPM phosphorylation status through a novel signalling pathway, PPM1D-CDC25C-CDK1-PLK1.

An increase of nucleolar number and size is observed in most cancers which has made nucleoli important markers of cancer prognosis^1^. For breast tumours especially, an increase in the nucleolar number is observed in high-grade tumours^2^,^3^. Initially, nucleoli were thought to be a sub-nuclear compartment solely devoted to ribosomal synthesis. However, recent reports show that the nucleolus is also involved in a number of other cellular events such as the regulation of M phase, cell cycle progression, cell proliferation, and stress response^4^. Nevertheless, the underlying basis for their structural integrity and abundance is still unclear^1^.

The assembly and disassembly of the nucleolus occurs during the cell cycle. It has been suggested that this process is dependent on the equilibrium between phosphorylation and dephosphorylation of several regulatory proteins. Formation of the nucleolus is regulated by the inactivation of CDK1-cyclin B, which occurs at the end of mitosis. The balance between CDK1 kinase and PP1 phosphatase activities has been shown to be one of the regulators of cell cycle-dependent assembly and disassembly of the nucleolus.

Nucleophosmin (also known as NPM, B23) is a nucleolar phosphoprotein involved in nucleoli assembly along with many cellular activities such as proliferation and growth suppression^5^. NPM has also been reported to be involved in processing and assembly of ribosomal biogenesis via its ability to (1) shuttle between nucleus and cytoplasm^6^,^7^ (2) bind nucleic acid^8^, and (3) transport maturing pre-ribosomal particles^9^. Depletion of NPM induces a distortion of the nucleolar formation^10^. NPM is phosphorylated at multiple sites by various kinases during different stages of the cell cycle. However, the link between NPM phosphorylation and its involvement in nucleolar formation is not fully understood. It was also reported that NPM is dysregulated in numerous solid and haematological malignancies^5^. In acute promyelocytic leukaemia (APL), anaplastic large cell lymphoma (ALCL), myelodysplastic syndrome (MDS) and acute myeloid leukaemia (AML), NPM is reported to form fusion proteins with ALK, RARα, and MLF1^5^.

*PPM1D* (Protein Phosphatase Magnesium-dependent 1, Delta, also known as PP2Cδ and Wip1), encoding a PP2C phosphatase, maps to 17q23.2. *PPM1D* was found to be up-regulated in a number of different carcinomas including breast and ovarian^11^,^12^,^13^,^14^,^15^. Gene amplification and overexpression of *PPM1D* are strongly associated with tumours displaying the luminal or HER2 phenotype suggesting a causal link^14^. PPM1D is a Ser/The protein phosphatase that is part of a negative feedback loop with p53 and is induced in a p53-dependent manner in response to DNA damage, dephosphorylating Ser15 on p53^16^,^17^,^18^. However, PPM1D also acts upon crucial signalling proteins such as ATM^19^, ATR^20^, Chk1^21^, Chk2^22^, p38^23^ and is known to inhibit p16^INK4a^ and ARF^24^. Thus, given PPM1D’s overexpression in most carcinomas and its various targets, it is likely to be involved in various important processes in cancer cells. A recent study of patients with invasive breast cancer found that amplification of PPM1D is also observed in p53 mutant tumours^14^. These facts suggest that p53-independent signalling cascades involving PPM1D exist in cancer cells.

Herein, we report the effect of PPM1D overexpression on the nucleolar formation and on the nucleolar protein NPM. We show that PPM1D overexpression induces an increase in nucleolar number regardless of p53 status. We also demonstrate for the first time that sequential phosphorylation of NPM on Thr199 and Ser4 by a novel PPM1D-CDC25C-CDK1-PLK1 pathway regulates nucleolar formation. These results demonstrate that PPM1D is a novel upstream regulator of the CDC25C-CDK1-PLK1-NPM signalling pathway and thus controls nucleolar formation.

## Results

### PPM1D suppression decreases nucleolar number

**Figure 1:**
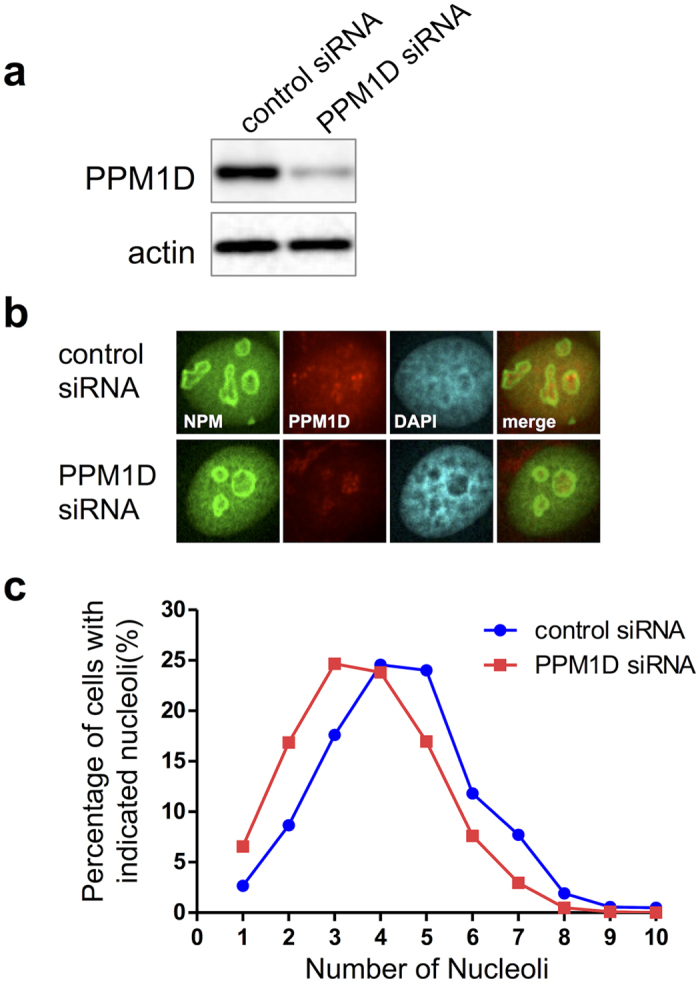
Nucleolar number was decreased by PPM1D knockdown. MCF-7 cells were treated with PPM1D siRNA or control siRNA for 48 h, and were solubilized with 1x sample buffer for (**a**) Western blotting or (**b**) Immunocytochemistry. (**a**) PPM1D proteins were analysed by using Western blotting with rabbit polyclonal anti-PPM1D. (**b**) The subcellular localizations of NPM and PPM1D. PPM1D siRNA- or control siRNA-treated MCF-7 cells were fixed and stained with rabbit polyclonal anti-PPM1D and mouse monoclonal anti-NPM. (**c**) The percentage of cells with indicated nucleoli in PPM1D siRNA- or control siRNA-treated MCF-7 cells are shown. For quantitative analysis of the nucleolar number, stained-NPM was used as an indicator of the nucleoli. Number of cells used in the analysis are shown in Table 1; from at least two independent experiments.

The human breast cancer cell line MCF-7 overexpresses PPM1D and harbours wild-type p53. Immunocytochemistry showed that PPM1D is localized either in the dense fibrillar components or in the fibrillar centre of the nucleolus, whereas NPM is localized in granular components. When the nucleoli were visualized with the marker NPM, we observed that PPM1D knockdown induced a significant decrease of the number of nucleoli in MCF-7 cells (Fig. 1a,b). In a quantitative comparison between nucleolar number and PPM1D overexpression, PPM1D-negative cells had an average of 3.7 nucleoli per cell while control siRNA-treated cells had an average of 4.4 nucleoli per cell (Fig. 1c; Table 1). The nucleolar number is known to increase at G1 and late G2 phase temporarily in accordance with breakdown of the nuclear envelop during cell cycle. Flow cytometry showed that PPM1D knockdown induced a slight increase of G1 phase, showing that the resulting reduction in nucleoli number had not occurred simply a result of differential cell cycle distribution (Table S1).

### PPM1D overexpression increases nucleolar number

**Figure 2:**
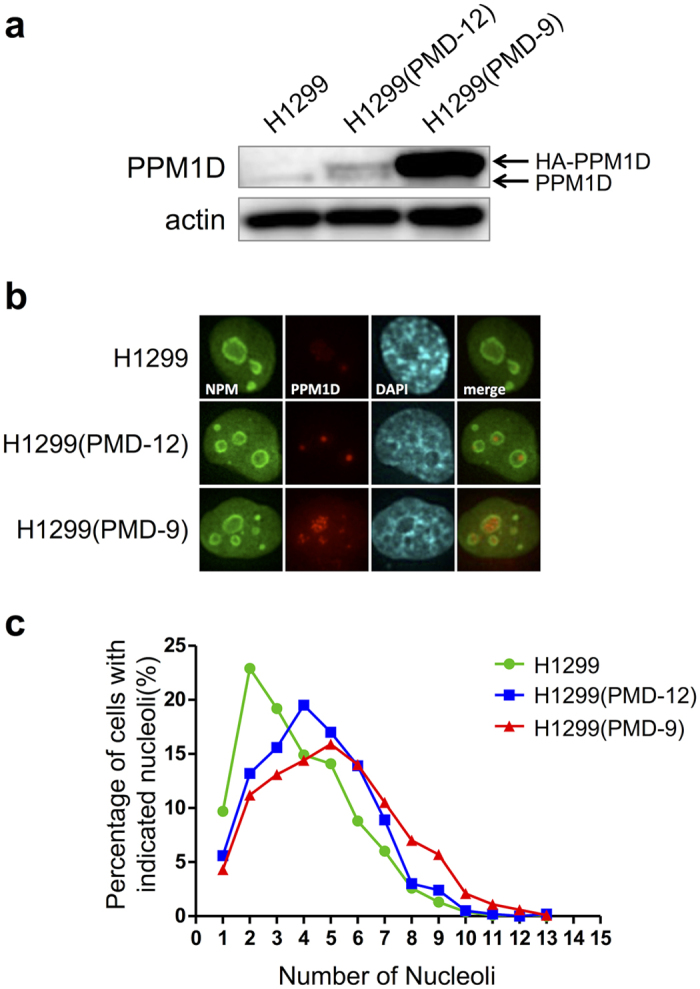
PPM1D overexpression induces an increase in nucleolar number. H1299(PMD-9) and H1299(PMD-12) were derived from H1299 in our laboratory and expressed HA-PPM1D stably. H1299 cells were solubilized with 1x sample buffer for Western blotting (**a**) or fixed with formaldehyde for Immunocytochemistry (**b**). (**a**) PPM1D proteins were analysed by using Western blotting with rabbit polyclonal anti-PPM1D. (**b**) The subcellular localizations of NPM and PPM1D. H1299 cells and clones were fixed and stained with rabbit polyclonal anti-PPM1D and mouse monoclonal anti-NPM. (**c**) The percentage of cells with indicated nucleoli in H1299 clones were shown. For the quantitative analysis of the nucleolar number, stained-NPM was used as an indicator of the nucleoli. Number of cells used in the analysis are shown in Table 2; from at least two independent experiments.

To further understand the mechanisms of PPM1D-induced nucleolar formation, we constructed H1299 cells that stably express HA-PPM1D (Fig. 2a,b, and S1). The human lung carcinoma cell line H1299 does not express p53 originally. Consistent with the results in MCF-7, PPM1D overexpression increased the nucleolar number in H1299 clones (Fig. 2b,c; Table 2). Flow cytometry confirmed that PPM1D overexpression induced a slight decrease of G1 phase (Table S1), showing that the increase in nucleoli was not as a result of cell cycle distribution (Table S2), in keeping with the results of the PPM1D knockdown experiment in MCF-7. Also, the nucleolar number was not affected by overexpression of catalytic inactive mutant PPM1D(D314A) (Fig. S2). Together, these results indicate that PPM1D regulates the nucleolar number in p53 wild type and p53 null cancer cells via its phosphatase activity.

Nucleoli size is also an important parameter in cytology. We therefore sought to determine whether PPM1D overexpression also increases the size of nucleoli. The size of nucleoli in H1299 was 50 μm^2^ (Table 3). In contrast, the size of nucleoli in PPM1D-overexpressing H1299(PMD-9) and H1299(PMD-12) cells^25^ was 61 and 52 μm^2^, respectively. PPM1D overexpression therefore resulted in a significant increase in nucleolar size. We concluded that PPM1D overexpression induces an increase not only in the nucleolar number, but also in nucleolar size.

### PPM1D knockdown induces a reduction of phosphorylated-NPM in p53 wild-type cancer cells

**Figure 3:**
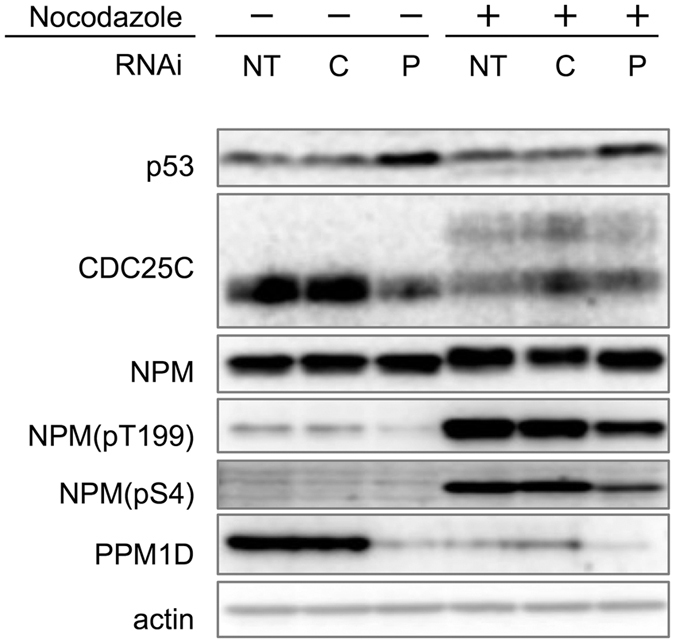
Reduction of phosphorylated-NPM by PPM1D knockdown in MCF-7 cells. Cells transfected with siRNA for 48 h were treated with 4 μg/ml nocodazole for 16 h. Cells were then harvested by mitotic shake off and analysed by Western blotting with mouse monoclonal anti-p53, rabbit monoclonal anti-CDC25C, mouse monoclonal anti-NPM, rabbit polyclonal anti-NPM(pT199), rabbit monoclonal anti-NPM(pS4), rabbit polyclonal anti-PPM1D and mouse monoclonal anti-actin. NT: no treatment, C: control siRNA-treated, P; PPM1D siRNA-treated.

To define the molecular mechanisms by which PPM1D acts, we next examined the post-translational modification state of the nucleolar protein NPM following PPM1D knockdown. MCF-7 was treated with nocodazole and harvested by mitotic shake off. PPM1D knockdown caused a decrease of phosphorylated-NPM at Thr199 and a significant decrease of phosphorylated-NPM at Ser4 (Fig. 3). It was noted that NPM protein level did not change by PPM1D knockdown in the condition with and without nocodazole treatment.

### The effect of mutations at NPM phosphorylation sites on nucleolar number

**Figure 4:**
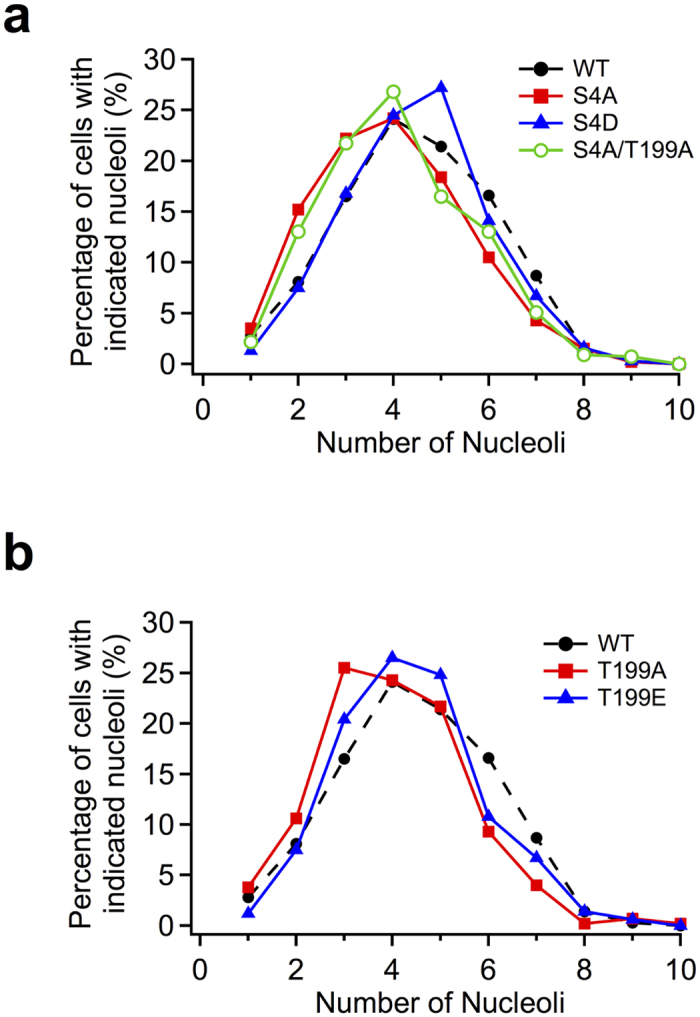
Effect of the phosphorylation sites of NPM on the nucleolar number. The distributions of nucleoli number among cells are shown as curves. MCF-7 cells were treated with endogenous NPM siRNA for 24 h, and, following medium change were transfected with HA-NPM mutants, at Ser4 and Ser4/Thr199 (a) and at Thr199 (b) fixed and stained with rabbit polyclonal anti-HA and mouse monoclonal anti-NPM. For quantitative analysis of the nucleolar number, stained-HA signal was used as indicators of the cells that were expressed with HA-tagged NPM mutants and counted nucleolar number only in the cells expressing HA-tagged NPM mutants. Number of cells used in the analysis are shown in Table 4; from at least two independent experiments.

NPM phosphorylation at Ser4 and Thr199 may play a role in maintaining or generating the structure of the nucleolus. To investigate the effect of the phosphorylation status at these NPM sites on the nucleolar number, we expressed HA-tagged NPM mutants in MCF-7 subsequent to endo-NPM knockdown and co-stained with NPM antibody and HA antibody (Fig. S3). All mutants localized to the nucleolus (Fig. S3). The cells with HA-NPM(WT) had an average of 4.5 nucleoli in one cell (Fig. 4, Table 4). The cells transfected with S4A or T199A mutants showed a decreased nucleolar number of 4.0. The cells transfected with NPM double mutant (S4A/T199A) exhibited a similar number of nucleoli with NPM(S4A) and NPM(T199A). The cells transfected with S4D or T199E phosphor-mimic mutants showed same number of nucleoli as WT. These results suggest that phosphorylation of NPM at Ser4 and Thr199 plays a role in determination of nucleolar number.

### Sequential phosphorylation of NPM by the CDK1-PLK1 cascade

**Figure 5:**
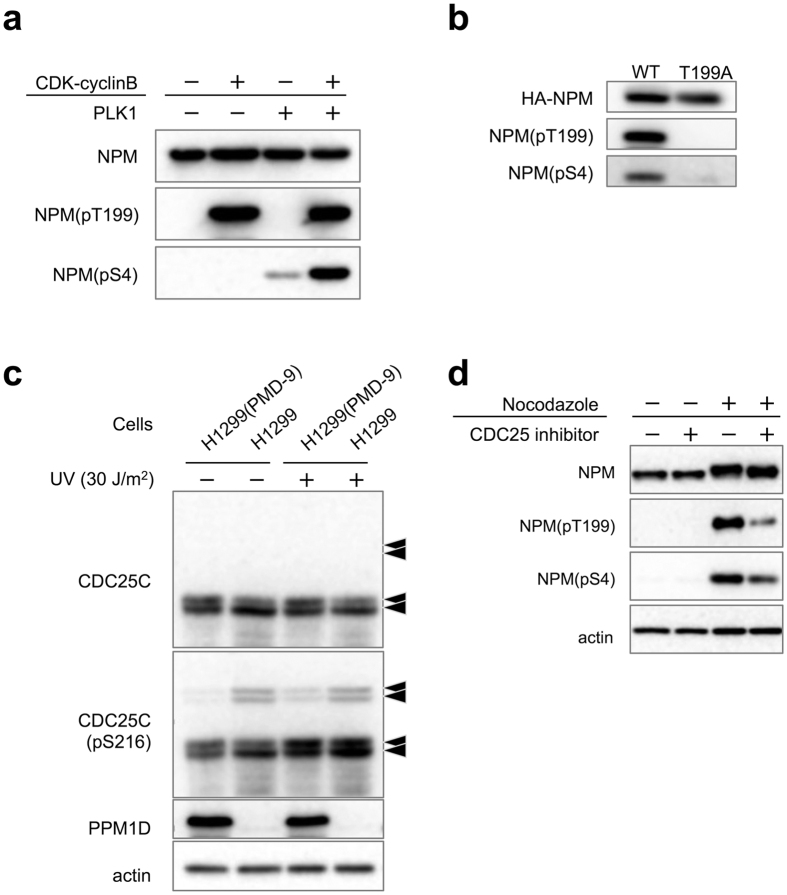
Signalling cascade in PPM1D-overexpressing cells. (**a**) *In vitro* sequential phosphorylation of NPM by CDK1-PLK1. His-NPM were expressed in *E. coli* and purified. 2 μg of His-NPM were incubated with 10 units of CDK1-cyclinB for 60 min at 30 °C, prior to incubation with 100 ng of PLK1 for 60 min at 30 °C. 2x sample buffer was added to stop the reaction. Samples were separated by SDS-PAGE and were subjected to Western blotting with same antibodies as in Fig. 3. (**b**) *In vivo* sequential phosphorylation of NPM. MCF-7 cells were transfected with either HA-NPM(WT) or HA-NPM(T199A) for 40 h then treated with 4 μg/ml nocodazole for 16 h. Lysates were then purified with mouse monoclonal anti-HA and analysed by Western blotting with polyclonal anti-HA and the same antibodies as Fig. 3. (**c**) Decrease of phosphorylated-CDC25C at Ser216 by PPM1D overexpression in H1299 clones. All arrows indicate CDC25C with different modification states. Cells were lysed 2 h after exposure to 30 J/m^2^ of UV radiation and analysed by Western blotting with rabbit monoclonal anti-CDC25C(pS216) and the same antibodies as Fig. 3. (**d**) Decrease of phosphorylated-NPM at Thr199 and Ser4 by CDC25 inhibitor in MCF-7. Cells were treated with 3 μM CDC25 inhibitor for 6 h and subsequently treated with 4 μg/ml nocodazole for 16 h. Cells were then harvested by mitotic shake off and analysed by Western blotting with mouse monoclonal anti-NPM, rabbit polyclonal anti NPM(pT199), rabbit monoclonal anti-NPM(pS4), and mouse monoclonal anti-actin.

Thr199 and Ser4 of NPM are phosphorylated by G2/M checkpoint kinase CDK1^26^ and polo-like kinase PLK1^27^, respectively. To explore the relationship between CDK1 and PLK1, we expressed His tagged NPM in *E. coli* and prepared active CDK1 and PLK1. An *in vitro* kinase assay showed that phosphorylated-NPM at Thr199 by CDK1 enhanced NPM phosphorylation at Ser4 by PLK1 as revealed by Western blotting with the phospho-specific NPM antibodies (Fig. 5a). We also confirmed this sequential phosphorylation of NPM by CDK1-PLK1 in MCF-7 cells (Fig. 5b). PPM1D exists in at least S phase as reported^28^, therefore this signalling cascade from PPM1D to NPM phosphorylation should be started before entering M phase.

### Suppression of CDC25C activity by PPM1D overexpression

PPM1D knockdown also increased the level of p53 protein and decreased CDC25C (Fig. 3). The latter is to be expected because CDC25C has been shown to be down-regulated by p53^29^. Taken together, therefore, it is possible that PPM1D overexpression induces an increase of CDC25C protein level via inhibition of p53. We also examined whether PPM1D overexpression induced an increase in CDC25C activity in H1299 cells. CDC25C is phosphorylated at Ser216 when CDC25C is expected to be inactive^30^. This phosphorylation occurs via interaction with various kinases throughout the cell cycle^31^. In H1299, the phosphorylation level of CDC25C at Ser216 increased compared with that of H1299(PMD-9) (Fig. 5c). Recombinant His-PPM1D (1–420) showed phosphatase activity against a synthetic phospho-peptide corresponding to residues surrounding Ser216 of human CDC25C, whereas phospho-peptides derived from the C-terminus of p53 were not dephosphorylated by PPM1D (Fig. S4). Furthermore, CDC25 inhibitor decreased the phosphorylation level of NPM at Thr199 and Ser4 (Fig. 5d). The latter observations suggest a PPM1D-CDC25C cascade independent of p53 status, proving that PPM1D up-regulates CDC25C activity by dephosphorylating Ser216. PPM1D overexpression thus up-regulates CDC25C via two mechanisms: 1) dephosphorylating Ser216 of CDC25C and 2) increasing protein level of CDC25C by inhibiting p53, both of which result in activation of CDK1 and PLK1. Our findings therefore support a model in which PPM1D controls the nucleolar structure independent from p53 status.

## Discussion

The dynamics of assembly and disassembly of the nucleolus are tightly regulated. Disassembly is a sequential process, starting at the beginning of mitosis, while assembly begins at telophase and progresses through a relatively long period of M phase^32^. The mechanism that governs disassembly of nucleoli in prophase is believed to be linked to repression of rDNA transcription, probably caused by CDK1-cyclin B-directed phosphorylation of components of the rDNA machinery^33^. In contrast, several groups reported that nucleoli assembly at the exit of M phase occurs independently of rDNA transcription^34^. It has been suggested that both processes are regulated through phosphoproteins by CDKs.

Cyclin-dependent kinase CDK1 is a protein that plays a major role in triggering the G2/M transition. Polo-like kinase PLK1 is expressed at a high level and functions at mitosis. PLK1 binds to a phosphorylated target via CDK1 through its polo-box domain (PBD) and phosphorylates the other site of same target protein^35^. In this report, we showed that phosphorylation of NPM by CDK1 including Thr199 induces sequential phosphorylation of Ser4 by PLK1. We also demonstrated that PPM1D is an upstream regulator of the CDC25C-CDK1-PLK1 cascade and controls the phosphorylation level of NPM. Furthermore, we show for the first time that sequential phosphorylation of NPM at Thr199 and Ser4 is important for regulating nucleolar formation.

The number and the size of nucleoli have provided useful prognostic markers in cancer^1^. For example, prostate cancer exhibited from 2.65–3.12 nucleoli whereas normal prostate exhibited 1.76 nucleoli^36^. It is also reported that benign breast tumors exhibited 2.96 and malignant tumors exhibited 4.0 nucleoli^37^. However, the means by which changes in nucleolar integrity downstream of signalling pathways contribute to a cell’s oncogenic activity remain poorly understood^1^. *PPM1D* is highly amplified and overexpressed in multiple human cancers^11^,^12^,^13^,^14^. It is also reported that PPM1D co-operates with H-rasV12, MYC and NEU1 in the transformation of wild type MEFs, confirming that *PPM1D* is a proto-oncogene^12^. PPM1D dephosphorylates cell cycle regulators such as ATM, ATR, Chk1, Chk2, p53, and p38^16^,^17^,^18^,^19^,^20^,^21^,^22^,^23^ and is thought to be involved in cancer progression or tumorigenesis thorough hyper-dephosphorylation. Determining the signal transduction pathways in cancer cells in which PPM1D participates is therefore a necessary step in the development of targeted therapy.

**Figure 6:**
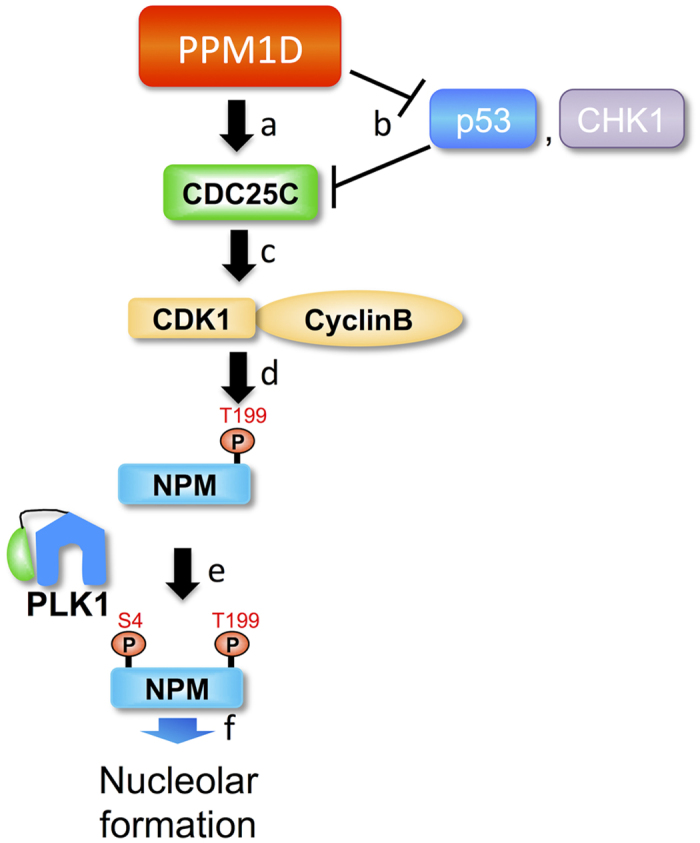
The signalling cascade responsible for regulating nucleolar formation. (**a**) PPM1D dephosphorylates CDC25C at Ser216. The site is phosphorylated when CDC25C is inactive, therefore PPM1D upregulates CDC25C activity. (**b**) PPM1D also dephosphorylates tumour suppressor protein p53. It is reported that DNA damage-induced down-regulation of CDC25C is mediated by p53, suggesting that PPM1D could also up-regulate CDC25C via p53 protein and/or other factors, such as CHK1. (**c**) Up-regulated CDC25C dephosphorylates and activates CDK1. (**d**) Activated CDK1 phosphorylates Thr199 of NPM. (**e**) PLK1 binds to Thr199-phosphorylated NPM through its PBD and induces phosphorylation of Ser4 on the same NPM molecule. It is suggested that phosphorylation of Thr199 of NPM by CDK1 enhances phosphorylation of Ser4 of NPM by PLK1. (**f**) These results suggest that sequential phosphorylation of NPM at Thr199 and Ser4 by a novel signalling cascade, PPM1D-CDC25C-CDK1-PLK1, influences nucleolar formation.

We observed that overexpression of PPM1D induced an increase in the nucleolar number regardless of p53 status. DNA damage-induced down-regulation of CDC25C is mediated by p53^29^. There is a possibility that PPM1D overexpression leads to an increase of CDC25C protein via inhibition of p53, which leads to activation of CDK1 and PLK1 that are upstream kinases of NPM. Also, as observed in p53 null cells, CDC25C at Ser216 could be a possible target of PPM1D. Phosphorylation of CDC25C at Ser216 is reported to inactivate CDC25C dephosphorylation activity^30^. Taken together, this suggests that PPM1D would up-regulate CDC25C activity regardless of p53 status. There is also a possibility of dephosphorylation of CDC25C was mediated by an indirect mechanism such as PPM1D-CHK1 pathway^21^. We also demonstrated for the first time that PPM1D up-regulated phosphorylation of Thr199 and Ser4 of NPM sequentially via the CDC25C-CDK1-PLK1 signalling cascade thereby modulating nucleolar formation (Fig. 6). While phosphorylation at Thr199 by CDK1 and Ser4 by PLK1 has been reported in separate studies^27^,^38^,^39^,^40^, an association between these sites has not previously been reported. Our results suggested that phosphorylation of Thr199 by CDK1 was important for phosphorylation of Ser4 by PLK1. Phosphorylation at Thr199 by CDK1 was suggested to be involved in dissociation of NPM from the centrosome, leading to centrosome duplication^40^, and in regulation of RNA binding activity of NPM^38^,^39^. Phosphorylation at Ser4 by PLK1 was reported to be involved in regulation of mitosis^27^. Other nucleolar factors, such as fibrillarin and nucleolin are involved in the nucleolar formation. Further studies are required to clarify the effect of PPM1D overexpression on such factors.

The available evidence indicates that morphological and functional changes in the nucleolus are also a consequence of increased demand for ribosome biogenesis^41^. However, phosphorylation of NPM at Thr199 is reported to inhibit ribosome biogenesis^39^. It seems likely, therefore, that the interaction between ribosome biogenesis and nucleolar number are more complex than previously suggested. Further study is required to examine the association of PPM1D on the nucleolar disassembly and assembly, and ribosomal biogenesis.

In this report, we have shown that the proto-oncogene PPM1D is an upstream regulator of a signalling cascade that controls nucleolar formation. This finding is significant in improving our understanding of the relationship between the mechanisms controlling tumorigenesis and nucleolar integrity. We also proved that knockdown of PPM1D reduced nucleolar number. Our findings therefore suggest that PPM1D is an attractive future target for cancer therapy.

## Methods

### Cell lines and materials

MCF-7 human breast carcinoma cells and H1299 human non-small-cell lung cancer carcinoma cells were obtained from ATCC (Rockville, MD, USA). H1299(PMD-9) and H1299(PMD-12) were derived from H1299 by transfection with HA-PPM1D expression plasmid in our laboratory^25^. The plasmids containing HA-NPM or an empty vector were used for transient expression of NPM. NPM variants were generated by the following oligonucleotides are used for mutations; 5′-CATGTCCATGGCATCTTCCAT-3′ for HA-NPM(S4A), 5′-TTGGCTGGAGCATCTCGTAT-3′ for HA-NPM(T199A), 5′-CATGTCCATATCATCTTCCAT-3′ for HA-NPM(S4D), and 5′-TTGGCTGGTT CATCTCGTAT-3′ for HA-NPM(T199E). All variants of NPM were cloned into the phCMV2 vector (Gene Therapy Systems, Inc., San Diego, CA, USA) for expression of the HA-tagged protein in mammalian cells. Rabbit polyclonal anti-PPM1D was prepared as previously described^18^. Other antibodies used include: Mouse monoclonal anti-p53 (DO-1) and either a rabbit or goat polyclonal anti-HA (HA-probe (Y-11); Santa Cruz Biotechnology, Santa Cruz, CA, USA); mouse monoclonal anti-actin (Ab-1; Calbiochem, La Jolla, CA, USA); mouse monoclonal anti-NPM (mouse anti-Nucleophosmin (FC-61991); Invitrogen, Carlsbad, CA, USA); rabbit monoclonal anti-pNPM(S4) (Phospho-NPM (Ser4) (D19C1) XP^®^ Rabbit mAb), rabbit polyclonal anti-pNPM(T199) (Phospho-NPM (Thr199) Antibody), rabbit monoclonal anti-pCDC25C(S216) (Phospho-CDC25C (Ser216)(63F9) Rabbit mAb) and rabbit monoclonal anti-CDC25C (cdc25C (5H9) rabbit mAb; all from Cell Signaling Technology, Beverly, MA, USA); and rat monoclonal anti-HA (Anti-HA affinity clone3F10; Roche Applied Science, Indianapolis, IN, USA). Secondary antibodies for Western blotting included: anti-mouse IgM HRP-linked antibody (KPL); anti-mouse IgG HRP-linked antibody (GE healthcare, Milwaukee, MI, USA); and anti-rabbit IgG HRP-linked antibody (New England Biolabs, Beverly, MA, USA). Secondary antibodies for immunocytochemistry included Alexa Fluor488 goat anti-mouse IgG and Alexa Fluor568 goat anti-rabbit IgG (Invitrogen). His-NPM were expressed in *E. coli* and purified. CDK1-cyclinB and PLK1 were obtained from New England BioLabs and EMD Millipore (Billerica, MA, USA) respectively. His-PPM1D(1-420) was expressed in *E. coli* and purified. Phosphorylated peptide analogues as substrate were as follows: CDC25C(210–212)216P: Ac-W-GLYRSPS(P)MPENLN-NH_2_, p53(373–386)378P: Ac-W-KGQSTS(P)RHKKLMFK-NH_2_, p53(381–393)392P: Ac-W-KKLMFKTEGPDS(P)D-OH.

### Cell manipulation

Cell lines were cultured in Dulbecco’s modified Eagles medium supplemented with 10% v/v foetal bovine serum with 100 units/ml of penicillin and 100 μg/ml of streptomycin in a humidified atmosphere of 5% CO_2_. When required, cells were treated 24 h prior to fixation with UV irradiation at 15 or 30 J/m^2^. Transfection of cells with HA-NPM or empty vector was performed with Lipofectamine 2000 (Invitrogen) under conditions as described by the manufacturer following endogenous NPM knockdown. 24 h after the transfection, cells were subject to immunocytochemistry and analysis of the nucleolar number and size (see immunofluorescence and quantification section). Target-specific siRNA duplexes were purchased from Invitrogen and the sequence information for both PPM1D and endogenous NPM were 5′-GAAGUGGACAAUCAGGGAAACUUUA-3′ and 5′-AUAUAUAGACCCUGAAGAUCUCGCG-3′, respectively. SiRNA duplexes were subject to a BLAST search against the human genome sequence to ensure specificity towards the targets by Invitrogen BLOCK-iTTM RNAi Designer. Transient transfection of siRNAs was carried out using Lipofectamine 2000 (Invitrogen) following the manufacturer’s instructions. Cells were assayed after 48 h of transfection. For each experiment, specific silencing was confirmed by immunoprecipitation and immunofluorescence. To synchronize cells at M phase, Cells transfected with siRNA for 48 h were treated with 4 μg/ml nocodazole for 16 h. Cells were then harvested by mitotic shake off and analysed by flow cytometry and Western blotting. For the experiments with CDC25 inhibitor, cells were treated with 3 μM CDC25 Phosphatase Inhibitor II (NSC663284, Santa Cruz Biotechnology) for 6 h and subsequently treated with 4 μg/ml nocodazole for 16 h. Cells were then harvested by mitotic shake off and analysed by Western blotting.

### Western blotting analysis

All cells were rinsed with ice-cold PBS and lysed in high salt lysis buffer (50 mM Tris-HCl, pH 7.5, 500 mM NaCl, 5 mM EDTA, 1% Triton X-100, 50 mM NaF, 10 mM sodium pyrophosphate, 25 mM β-glycerophosphate, 1 mM sodium orthovanadate, 1 mM sodium molybdate, 1 mM p-Amidinophenyl Methanesulfonyl Fluoride) or sampled with 1x sample buffer (50 mM Tris-HCl, pH 6.8, 10% Glycerol, 2% SDS, 6% 2-mercaptoethanol). Equivalent amounts of total cellular protein were separated by SDS-PAGE and transferred to polyvinylidene difluoride membranes. Proteins were detected by enhanced chemiluminescence with the above antibodies.

### Flow cytometry

Cells were fixed with 70% ethanol, harvested and resuspended in PI/RNase staining buffer (BD Pharmingen, San Diego, CA, USA). DNA cell cycle analysis was performed by flow cytometry (FACSCalibur, FACStation ver 1.1) and analysed using Flow Jo 7.5 software (Tree Star Inc., Ashland, OR, USA).

### Immuno-purification of HA-tagged NPM mutants

HA-tagged NPM mutants were immuno-purified with anti-HA (3F10) antibody using protein G agarose beads from cell lysate and followed by Western blotting with the above antibodies.

### Immunofluorescence studies and quantification

To visualize nucleoli content, cells were cultured on glass coverslips and incubated for 48 h or 72 h. Cells were fixed with 3.5% formaldehyde for 15 min, washed in PBS, permeabilized with 0.2% Triton X-100/PBS for 15 min, and blocked with 10% FBS/PBS for 1 h. After incubation with primary antibodies, rabbit polyclonal anti-PPM1D and mouse monoclonal anti-NPM (mouse anti-Nucleophosmin (FC-61991)), cells were stained with the secondary antibodies Alexa Fluor488 goat anti-mouse IgG and Alexa Fluor568 goat anti-rabbit IgG, along with 4′,6-diamidino-2-phenylindole. Cells were then observed by fluorescence microscopy (BZ-9000, KEYENCE, Osaka, Japan). For quantitative analysis of the nucleolar number, stained-NPM was used as an indicator of the nucleoli. Nucleolar number of nucleoli were analysed by hand with a counter and data are given as mean. Significance was assessed using the Wilcoxon rank-sum test. Significance was established at *p* < 0.001. For the analysis of area of total nucleoli, data were obtained by copying the image of nucleoli for each cell on to tracing paper after which they were cut out and weighed. Total nucleoli from 105 to 215 cells were cut out, and their weight converted into area. Data regarding nucleolar size of nucleoli are given as mean ± standard error. Significance was established at *p* < 0.1 from three independent experiments using the Student’s t-test.

### *In vitro* kinase assay

Two micrograms of His-NPM were incubated with 10 units of CDK1-cyclinB for 60 min at 30 °C, prior to incubation with 100 ng of PLK1 for 60 min at 30 °C. 2x sample buffer (100 mM Tris-HCl, pH 6.8, 20% glycerol, 4% SDS, 12% 2-mercaptoethanol) was added to stop the reaction. Samples were separated by SDS-PAGE and were subjected to Western blotting with the above antibodies.

### *In vitro* phosphatase activity assay

Phosphatase activity was assayed by measuring the released free phosphate using the BIOMOL GREEN Reagent (BIOMOL, Plymouth Meeting, PA, USA) following the manufacturer’s protocol. The amount of phosphatase released was calculated using a phosphate standard curve. All assays were carried out in Tris buffer (50 mM Tris–HCl pH 7.4, 50 mM NaCl, 30 mM MgCl_2_, 0.1 mM EGTA, 0.02% 2-mercaptoethanol) by incubation with phosphopeptides and His-PPM1D420 (4, 10, 20 nM) for 25 min at 30 °C.

## Additional Information

**How to cite this article**: Kozakai, Y. *et al*. PPM1D controls nucleolar formation by up-regulating phosphorylation of nucleophosmin. *Sci. Rep.* **6**, 33272; doi: 10.1038/srep33272 (2016).

## Supplementary Material

Supplementary Information

## Figures and Tables

**Table 1:**
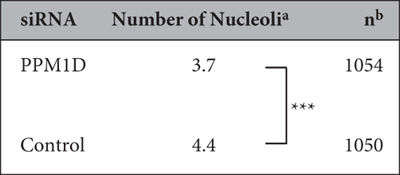
PPM1D knockdown induces a decrease in nucleolar number.

^a^Data were analysed by hand with a counter and data represent the mean. Significance was analysed by using the statistical Wilcoxon rank-sum test. ***p < 0.001. ^b^Total cell numbers in PPM1D siRNA and control siRNA treated cells; from at least two independent experiments.

**Table 2:**
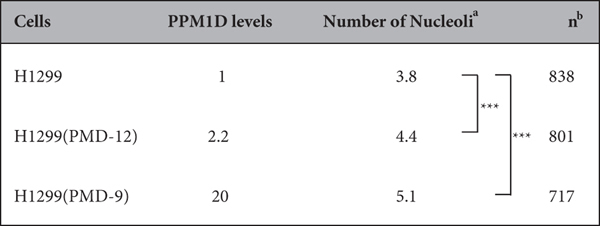
PPM1D overexpression induces an increase in nucleolar number.

^a^The number of nucleoli in each cell was counted manually and data represent the mean. Significance was analysed by using the statistical Wilcoxon rank-sum test. ***p < 0.001. ^b^Total cell number using the analysis in at least two independent experiments.

**Table 3:**
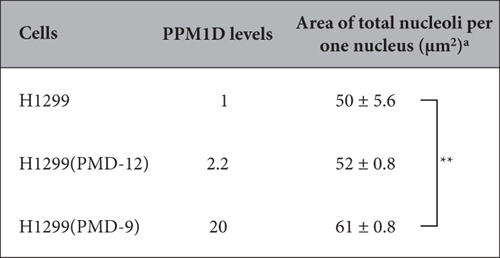
PPM1D overexpression induces an increase in nucleolar size.

^a^Data were analysed by copying the image of nucleoli for each cell on tracing paper and weighing them. Total nucleoli from 101 to 215 cells were cut out, and their weight converted into area. Data represent the mean ± standard error. **Significance was established at *p* < 0.1 from three independent experiments.

**Table 4:**
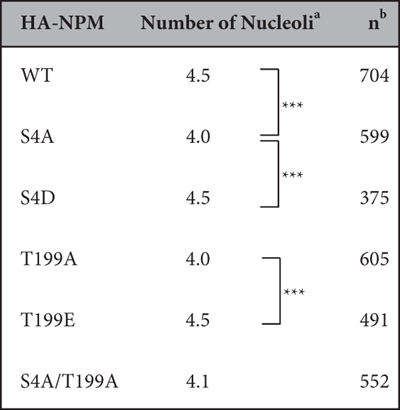
Effect of the phosphorylation sites of NPM on the nucleolar number.
