# Supplementary material for: PPM1D controls nucleolar formation by up-regulating phosphorylation of nucleophosmin
Source: Sci Rep. 2016 Sep 13;6:33272. doi: 10.1038/srep33272 (PMC5020408; doi:10.1038/srep33272)
Supplement: Supplementary Information [file srep33272-s1.pdf]

Supplementary Information

PPM1D controls nucleolar formation by up-regulating phosphorylation of nucleophosmin

Yuuki Kozakai[1], Rui Kamada[1**], Junya Furuta[1], Yuhei Kiyota[1], Yoshiro Chuman[1], Kazuyasu Sakaguchi[1*]**

[1] Laboratory of Biological Chemistry, Department of Chemistry, Faculty of Science, Hokkaido University, Sapporo, Japan.

[*] Corresponding Author:
Kazuyasu Sakaguchi, PhD
Laboratory of Biological Chemistry
Department of Chemistry
Faculty of Science
Hokkaido University
Phone: +81-11-706-2698
Fax: +81-11-706-4683
E-mail: kazuyasu@sci.hokudai.ac.jp

**These authors contributed equally to this work.

Supplementary Table S1.

PPM1D knockdown	-	+
G2/M	20.1	19.5
S	20.0	15.7
G1	55.0	61.5
subG1	4.9	3.2

Cells were transfected with siRNA for 48 h. Cells were fixed with 70% ethanol, harvested and resuspended in PI/RNase staining buffer. DNA cell cycle analysis was performed by flow cytometry using FlowJo 7.5 software.

Supplementary Table S2.

Cells	H1299(PMD-9)	H1299
G2/M	15.7	16.8
S	48.0	41.7
G1	33.7	39.9
subG1	2.6	1.7

H1299 and H1299(PMD-9) were analysed by Flow cytometry. Cells were fixed with 70% ethanol, harvested and resuspended in PI/RNase staining buffer. DNA cell cycle analysis was done by flow cytometry using FlowJo 7.5 software.

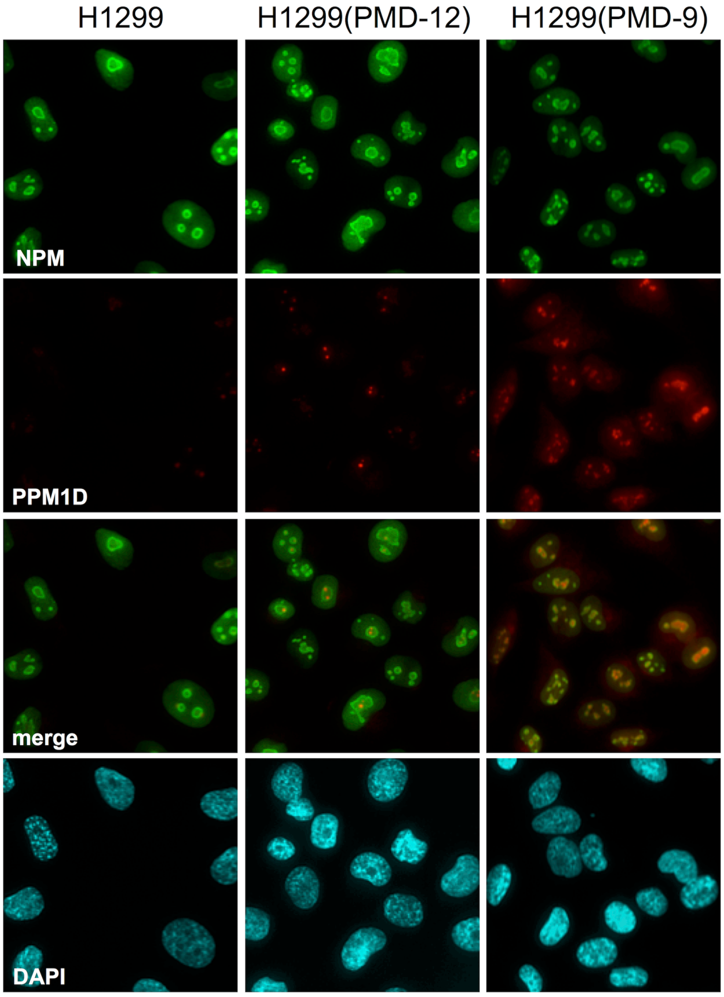

Supplementary Figure S1. H1299(PMD-9) and H1299(PMD-12) were derived from H1299 in our laboratory and expressed HA-PPM1D stably. H1299 cells and clones were fixed and stained with rabbit polyclonal anti-PPM1D and mouse monoclonal anti-NPM.

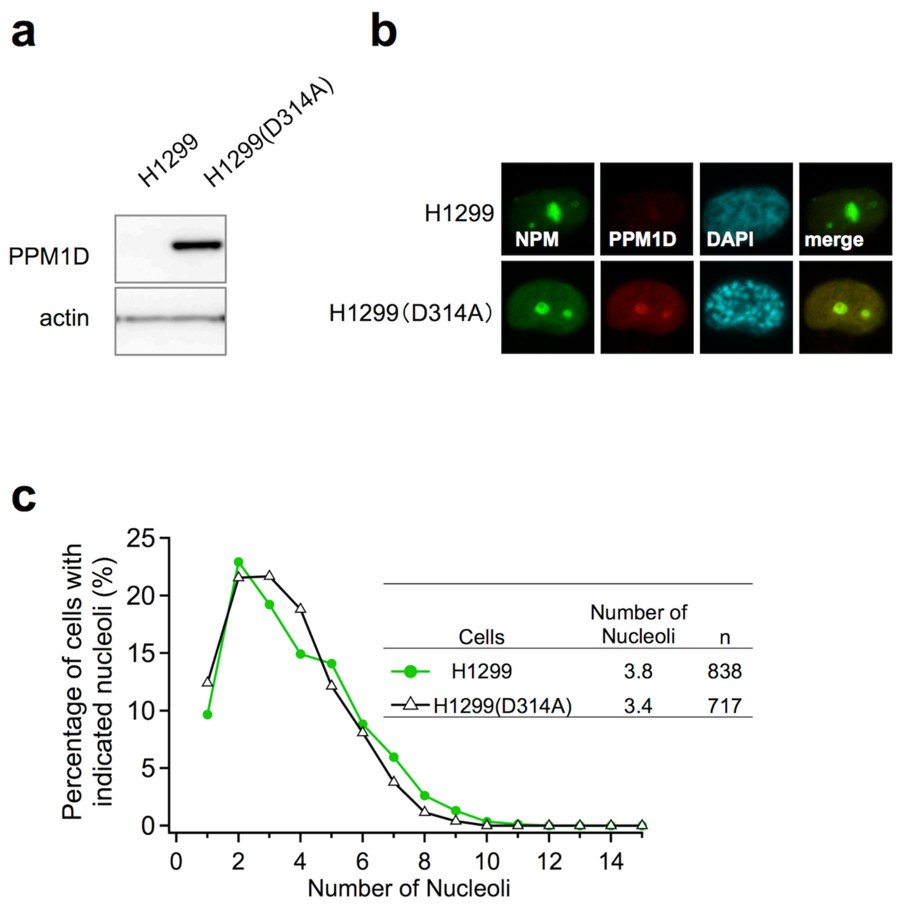

Supplementary Figure S2. H1299(D314A) cells were derived from H1299 in our laboratory and stably expressed HA-PPM1D(D314A), which is inactive mutant of PPM1D. H1299 and H1299(D314A) cells were analysed by Western blotting (a) or fixed with formaldehyde for Immunocytochemistry (b). (c) The percentage of cells with indicated nucleoli in H1299 clones were shown. For the quantitative analysis of the nucleolar number, stained-NPM was used as an indicator of the nucleoli. Data represent the mean and *n* values represent the number of cells used in the analysis from at least two independent experiments.

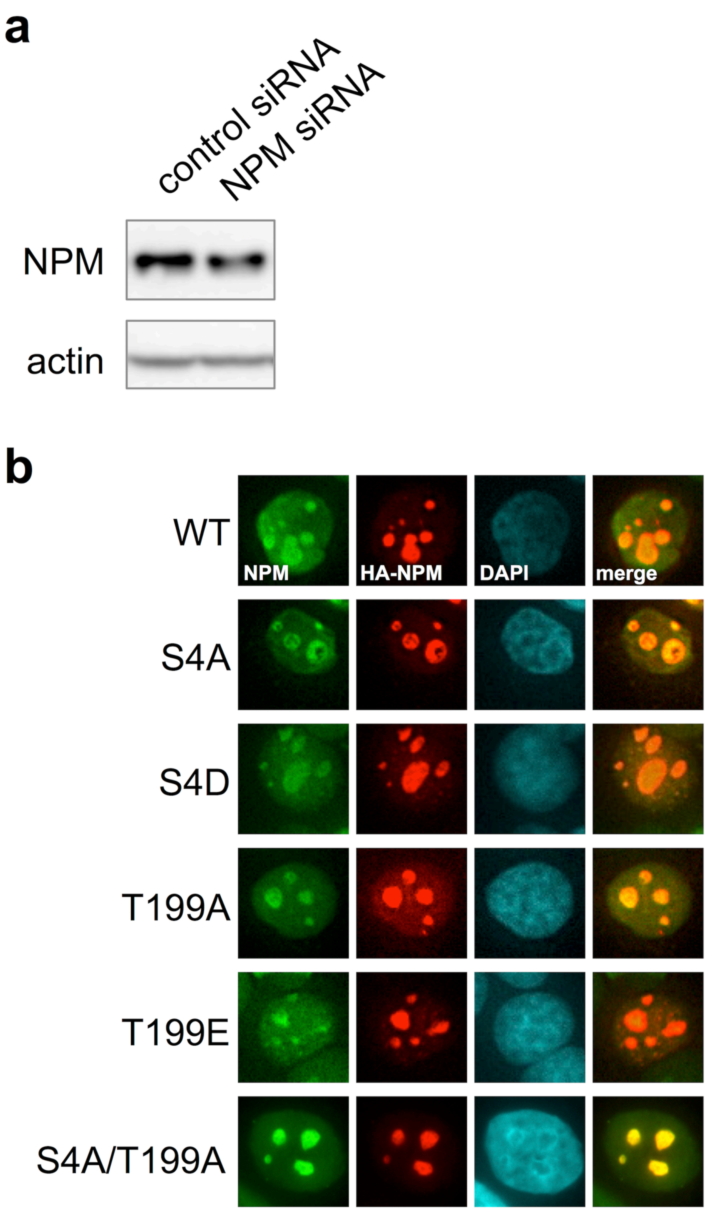

Supplementary Figure S3. (a) MCF-7 cells were treated with endogenous NPM siRNA for 24 h and NPM proteins were analysed by Western blotting with mouse monoclonal anti-NPM. (b) MCF-7 cells were treated with endogenous NPM siRNA for 24 h, and, following medium change, were transfected with HA-NPM mutants, and fixed and stained with rabbit polyclonal anti-HA and mouse monoclonal anti-NPM for visualizing transfected HA-NPM and whole NPM, respectively.

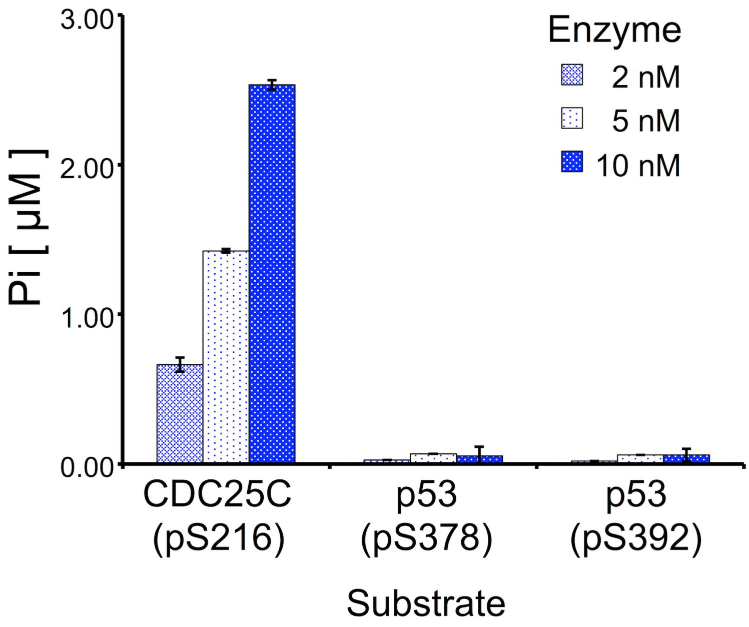

Supplementary Figure S4. Phosphatase activity was assayed by measuring the released free phosphate by BIOMOL GREEN Reagent following the protocol provided by BIOMOL (see Methods). All assays were carried out in Tris buffer by incubation with phosphopeptides (40 µM) and His-PPM1D420 (2, 5, 10 nM) for 25 min at 30°C.
